# Influence of the relative age effect on children’s scores obtained from the Canadian assessment of physical literacy

**DOI:** 10.1186/s12889-018-5895-6

**Published:** 2018-10-02

**Authors:** Caroline Dutil, Mark S. Tremblay, Patricia E. Longmuir, Joel D. Barnes, Kevin Belanger, Jean-Philippe Chaput

**Affiliations:** 1 School of Human Kinetics, Faculty of Health Sciences, University of Ottawa, Ottawa, ON K1N 6N5 Canada; 2 Healthy Active Living and Obesity Research Group, Children’s Hospital of Eastern Ontario Research Institute, 401 Smyth Road, Ottawa, ON K1H 8L1 Canada

**Keywords:** Movement skills, Health behaviours, Motivation, Physical activity, Month of birth bias, Pediatric

## Abstract

**Background:**

Age grouping by the imposition of a cut-off date, common in sports and education, promotes a relative age difference that is associated with developmental advantages for children who are born on the “early side” of the cut-off date and disadvantages to those born later in the same year, which is known as the relative age effect (RAE) bias. Acquiring an adequate level of physical literacy is important for children to remain active for life. The Canadian Assessment of Physical Literacy (CAPL) is an assessment protocol that encompasses measures in the domains of children’s Daily Behaviours, Physical Competence, Motivation and Confidence, and Knowledge and Understanding. The purpose of this study was to ascertain whether the CAPL scores were susceptible to the RAE, which could affect our interpretation of the CAPL findings.

**Methods:**

This cross-sectional study examined if scores obtained in the CAPL (i.e., the four domains individually and the total CAPL score) were susceptible to the RAE in children aged 8 to 12 years and, if so, which physical competence assessments (movement skills, cardiorespiratory, strength, muscular endurance, flexibility, and body composition measurements) were more susceptible. Participants (*n* = 8233, 49.8% boys) from the Royal Bank of Canada–CAPL Learn to Play project from 11 sites in seven Canadian provinces were tested using the CAPL protocol.

**Results:**

Among boys and girls, the RAE was significantly associated with two and three of the four domain scores, respectively, after controlling for covariates. However, effect sizes were negligible for the comparisons between quarters of the year and physical literacy domains and overall scores. For the main effect of the relative age, boys and girls born in the first three months of the year were taller (*F*(3, 4074) = 57.0, *p* < 0.001, ƒ^2^ = 0.04 and *F*(3, 4107) = 58.4, *p* < 0.001, ƒ^2^ = 0.04, respectively) and demonstrated greater muscular strength (*F*(3, 4037) = 29.2, *p* < 0.001, ƒ^2^ = 0.02 and *F*(3, 4077) = 25.1, *p* < 0.001, ƒ^2^ = 0.02, respectively) compared with those born later in the same year.

**Conclusions:**

Collectively, our results suggest that the RAE bias is mainly negligible with regard to the domain scores and overall CAPL scores in this large sample of children.

**Electronic supplementary material:**

The online version of this article (10.1186/s12889-018-5895-6) contains supplementary material, which is available to authorized users.

## Background

Physical literacy is a concept that has gained momentum globally; it is defined by the International Physical Literacy Association as “the motivation, confidence, physical competence, knowledge and understanding to value and take responsibility for engagement in physical activity for life” [1]. The goal of physical literacy (i.e., lifelong engagement in physical activity) is an attractive idea, especially when considering how critical physical activity is for children’s health and well-being [2]. Many countries have adopted and included the physical literacy construct in their education system, and sports governing bodies have also followed suit in their athlete development programs [3–5].

In response to the need to assess physical literacy in children, the Canadian Assessment of Physical Literacy (CAPL) was recently developed and validated [6, 7]. The CAPL is a comprehensive measurement tool that encompasses many aspects within the four relevant domains of physical literacy (Physical Competence, Daily Behaviour, Motivation and Confidence, and Knowledge and Understanding) and provides an overall physical literacy score for 8- to 12-year-old children [6, 7]. The CAPL’s four domains are aligned with the current physical literacy definition [1, 6, 7].

In sports and education systems, children are commonly grouped by age as an administrative strategy in order to provide age-adjusted competition and learning opportunities. However, age grouping by the imposition of a cut-off date promotes a relative age difference that often leads to developmental advantages for children who are born on the “early side” of the cut-off date. These differences are known as the relative age effect (RAE) bias [8–11]. Indeed, those who are disadvantaged by the RAE are underrepresented at the elite and professional level in many sports [10], are at greater risk of having inferior grades in school [12–15], demonstrate lower levels of self-efficacy [16], display poorer mental health coping mechanisms [17], are at increased risk of needing special education support [18], are at greater risk of dropping out from sports [19, 20], and are at greater risk of being diagnosed with attention deficit hyperactivity disorder [21].

Studies on the RAE have also expanded to the testing of fitness, fundamental movement and developmental skills, where children are often compared using normative data that group children into rounded-down age bands (e.g., where a 9.9-year-old may be compared to a 9.1-year-old in an age band for all 9-year-old children). It is no surprise that an association between fitness, developmental and fundamental movement skill assessments and the RAE have been found, since this form of age grouping is analogous to the cohort grouping seen in sports and education systems [11, 22–24]. However, no study to date has examined the RAE in the context of physical literacy, a construct that simultaneously takes into account affective, cognitive, behavioural, and physical measurements. The present study was conducted to address this knowledge gap, and to assess the magnitude of the RAE using a comprehensive physical literacy assessment protocol.

The CAPL is scored by separating children into rounded-down age bands that span a minimum of a full year. Furthermore, if education systems and sports governing bodies are delivering physical literacy programs, school entry and sports cut-off dates become important factors in children’s acquisition of physical literacy skills, especially considering that cut-off dates are similar across many sports and education systems in many developed countries [10].

Thus, the present study aimed to examine the patterns of association between month of birth (relative age) and physical literacy assessment scores (i.e., individual domain scores and the total CAPL scores) in children aged 8 to 12 years. We also examined the magnitude of the RAE in the different components within the physical competence domain (i.e., movement skills, cardiorespiratory fitness, strength, muscular endurance, flexibility, and body composition measurements). We hypothesized that children born earlier in the year would score higher on physical literacy components than those born later in the same calendar year.

## Methods

### Participants

The Royal Bank of Canada (RBC) CAPL Learn to Play project was a cross-sectional study comprising a large number of Canadian children between 8 and 12 years of age. The aim was to recruit 12,500 children total from 11 Canadian sites: Victoria, British Columbia; Lethbridge, Alberta; Calgary, Alberta; Winnipeg, Manitoba; North Bay, Ontario; Windsor, Ontario; Ottawa, Ontario; Trois-Rivières, Québec; Halifax, Nova Scotia; Antigonish, Nova Scotia; and Charlottetown, Prince Edward Island. Although this was a convenience sample, testing sites were instructed to recruit children in a variety of settings (e.g., elementary schools, after-school programs and community centres) from urban, suburban and rural areas in and around their region, while also trying to ensure a balanced representation of high-, medium-, and low-income communities. Participants were tested by trained appraisers between February 2014 and February 2017. A parent (or legal guardian) provided written consent for participation in the study and completed a screening form indicating that their child had no known limitations for physical activity, including maximal effort exercise. Children also gave assent to partake in this assessment, and the Ethics review boards at each participating institution also approved the protocol.

### Study protocol

Physical literacy was assessed using the CAPL protocol. Longmuir and colleagues [7] have published a detailed explanation of the CAPL protocol, including its validity. The CAPL is also available online (www.capl-eclp.ca), and includes a detailed manual, training videos, and other relevant information that can be accessed or downloaded, in either English or French, for free [25]. The CAPL instrument measures, which are consistent with the current definition of physical literacy by the International Physical Literacy Association, assess each of the four domains of physical literacy (Physical Competence, Daily Behaviour, Knowledge and Understanding, and Motivation and Confidence), and provide an overall composite physical literacy score (i.e., total CAPL score) [6, 7].

A Delphi expert panel process was used to inform the CAPL scoring system. The total CAPL score (maximum of 100 points) is a composite sum of the scores obtained in the four domains, where both the Physical Competence and the Daily Behaviour domains are more heavily weighted (32 points each) than the Knowledge and Understanding and the Motivation and Confidence domains (18 points each) (see Additional file 1) [6, 7]. For more details on Canada’s physical literacy consensus statement, process, outcomes, and normative data, see Tremblay and colleagues [26, 27]. A short explanation of each domain is provided below.

### Physical competence domain

The aim of the Physical Competence domain is to test children’s physical core competencies to partake in physical activities by assessing their physical fitness, movement skills, and body composition. The score for this domain is composed of objective measurements of body composition (body mass index [BMI] *z*-score [28] and waist circumference [WC] [29]), cardiorespiratory fitness (Progressive Aerobic Cardiovascular Endurance Run [PACER] shuttle run) [30], muscular strength (grip strength) [29], muscular endurance (timed plank test) [31], flexibility (sit-and-reach) [29], and movement skills performance (Canadian Agility and Movement Skill Assessment [CAMSA]) (see Additional file 2) [32].

### Daily behaviour domain

The Daily Behaviour domain contains three components: average daily step counts measured via pedometer worn for seven consecutive days, self-reported sedentary time, and self-reported moderate to vigorous physical activity. Pedometer data criteria were established as follows: (i) step counts between 1000 and 30,000 steps daily [33]; (ii) minimum wear time of 10 h daily [34]; and (iii) at least three days of valid data that meet both aforementioned criteria [35]. The two other components were subjectively assessed via questionnaire, where children were asked to recall how many days in the past week they had engaged in a total of 60 min or more of moderate to vigorous physical activity, and to self-report their daily screen time habits [25]. For more details on the sedentary behaviour assessment, see Saunders and colleagues [36].

### Knowledge and understanding domain

The Knowledge and Understanding domain was assessed using a questionnaire that was designed to test aspects of healthy behaviour and the knowledge level that is expected based on Canadian physical and health education curricula (for grades 4, 5, and 6) across all provinces/territories [6]. The questions evaluate children’s knowledge and understanding of the Canadian Physical Activity and Sedentary Behaviour Guidelines for Children and Youth (http://csepguidelines.ca/children-and-youth-5-17/), related terms, definition of health, recommended safety equipment to partake in certain physical activities and sports, and a basic understanding of how movement skills can be improved [6, 7, 37].

### Motivation and confidence domain

The Motivation and Confidence domain, which was assessed via questionnaire, evaluated children’s self-perceived benefits and barriers for physical activity, self-perceived activity and skill levels compared to peers, and their adequacy in and predilection for physical activity [6, 7, 38]. The questions on physical activity barriers and benefits were based on the published scales developed by Garcia and colleagues [39]. Two thirds of this domain score were attributed to children’s responses to the adequacy and predilection subscales of the Children’s Self-Perceptions of Adequacy in and Predilection for Physical Activity (CSAPPA) questionnaire [40].

### Analytic sample

**Fig. 1:**
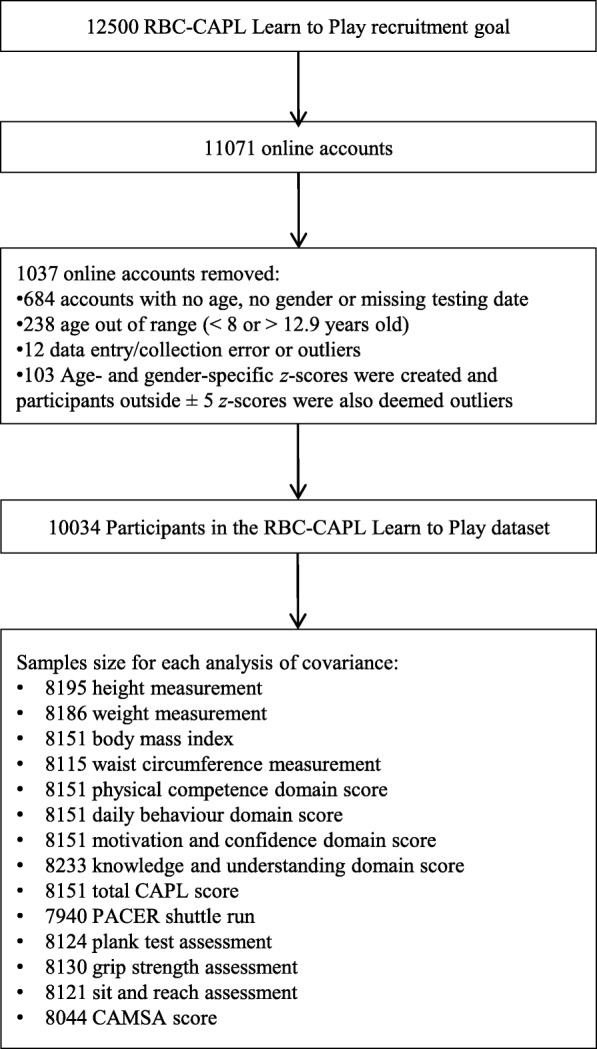
Sample sizes from recruitment goal to the number of participants in each analysis. Abbreviations: *CAMSA* Canadian Agility and Movement Skill Assessment, *CAPL* Canadian Assessment of Physical Literacy, *PACER* Progressive Aerobic Cardiovascular Endurance Run, *RBC* Royal Bank of Canada.

A flow diagram of the samples used in the present study is shown in Fig. 1. A little over 11,000 online accounts were created for the RBC–CAPL Learn to Play project; of those, any accounts that were outside the CAPL validated age group or that were missing key information were excluded. Any participants with raw scores less than quartile 1- (1.5 x the interquartile range) or greater than quartile 3+ (1.5 x the interquartile range) were deemed outliers and removed from the dataset. Age- and gender-specific *z*-scores were created, and participants outside ±5 *z*-scores were also deemed outliers and removed. Participants were also excluded from the analyses if they (or a parent) reported a medical condition or disability that would likely influence the CAPL assessment scores (*n* = 36) (e.g., broken limb in a cast, Down syndrome, autism, and those who reported acute injury on the day of testing). For the purpose of the present paper, those with birth month missing were ineligible and removed (n = 3). For all the analyses, those with incomplete scores for the dependent variable of interest were excluded. However, for the domain scores, it is possible to calculate a domain score if one assessment is missing from the domain, so those participants remained in the analytic samples [6, 7, 25]. Descriptive characteristics of children who were excluded did not differ from those who were included in the present analyses.

### Statistical analysis

In the present study, the independent variable was the relative age of the children based on month of birth and school entry cut-off date. The dependent variables were the scores obtained in each individual domain of CAPL as well as the overall CAPL score. Since the Physical Competence domain assesses many different components that may be unequally affected by the RAE, these dependent variables were also assessed individually. Covariates used for this study included age (in whole year), the testing month, the testing sites (geographic location based on the specific testing site), and children’s BMI *z*-scores (when relevant to the dependent variable). In order to examine the associations between the RAE bias and the scores obtained on physical literacy, children were stratified into quarters of the year based on their birth month and the annual school entry cut-off date. For all the sites except one, the annual school entry cut-off date is December 31, so for those sites children born in January, February, and March were assigned to quarter 1; children born in April, May, and June were assigned to quarter 2; etc. For the Trois-Rivières site in the province of Québec, the school entry cut-off date is October 1; therefore, we adjusted the quarter grouping for those participants (*n* = 42) accordingly for all analyses of covariance (ANCOVA) (i.e., children born in October, November, and December were assigned to quarter 1; children born in January, February, and March were assigned to quarter 2; etc.). Data were tested for assumptions of normality, linearity, and homogeneity of variance. As a result, no transformations were necessary.

As expected, our sample displayed an interaction effect for gender in the relationship between relative age and performance in CAPL assessments; boys and girls were therefore analyzed separately. However, no interaction was found for age (in whole years) for the same relationship; thus, all ages were analyzed together. We performed ANCOVAs, controlling for the above-mentioned covariates (age, testing month, testing sites and BMI *z*-scores [when relevant to the dependent variable]), with a Bonferroni post-hoc test to identify individual differences in mean scores per quarters. An alpha level of < 0.05 was set to establish statistical significance. Finally, to assess the practical or clinical significance of our findings, we calculated Cohen’s ƒ^2^ [41] from the partial η^2^ to interpret the effect sizes for our main effect (relative age in quarters). We interpreted the effect sizes according to Cohen’s guidelines [41], i.e., ƒ^2^ ≥ 0.02, ƒ^2^ ≥ 0.15 and ƒ^2^ ≥ 0.35 representing small, medium and large effect sizes, respectively. All statistical analyses were conducted using IBM SPSS Statistics for Windows, version 24 (IBM Corp., Armonk, NY, USA).

## Results

Tables 1 and 2 show the descriptive characteristics of our participants (49.8% boys). Birth months divided into quarters were not distributed evenly; almost 30% of the sample was born in the months of July, August, and September. However, our sample was representative of the Canadian birth distribution [42] according to a Chi-square goodness-of-fit test.

**Table 1:** Descriptive characteristics for children who participated in the RBC-CAPL study

	All		Boys		Girls	
	n	Mean (SD) or %	n	Mean (SD) or %	n	Mean (SD) or %
Age (y)	8233	10.6 (1.2)	4100	10.6 (1.2)	4133	10.6 (1.2)
Height (cm)	8195	144.1 (9.8)	4081	144.0 (9.6)	4114	144.3 (10.1)
Weight (kg)	8186	40.0 (11.5)	4076	40.0 (11.5)	4110	40.1 (11.4)
BMI (kg/m^2^)	8151	19.0 (3.8)	4059	18.9 (3.854)	4092	19.0 (3.727)
WC (cm)	8115	67.3 (10.8)	4039	67.4 (11.0)	4076	67.1 (10.5)
Sites (province):	8233	100.0	4100	100.0	4133	100.0
Antigonish (NS)	840	10.2	410	10.0	430	10.4
Calgary (AB)	1126	13.7	564	13.8	562	13.6
Charlottetown (PEI)	456	5.5	230	5.6	226	5.5
Halifax (NS)	648	7.9	321	7.8	327	7.9
Lethbridge (AB)	900	10.9	444	10.8	456	11.0
North Bay (ON)	966	11.7	457	11.1	509	12.3
Ottawa (ON)	619	7.5	292	7.1	327	7.9
Trois-Rivières (QC)	42	0.5	27	0.7	15	0.4
Victoria (BC)	425	5.2	225	5.5	200	4.8
Windsor (ON)	1108	13.5	578	14.1	530	12.8
Winnipeg (MB)	1103	13.4	552	13.5	551	13.3

*AB* Alberta, *BC* British Columbia, *BMI* body mass index, *MB* Manitoba, *NS* Nova Scotia, *ON* Ontario, *PEI* Prince Edward Island, *QC* Quebec, *RBC* Royal Bank of Canada, *SD* standard deviation, *WC* waist circumference

**Table 2:** Comparison of our sample’s birth distribution with Canadian live birth data between 2002 and 2008

Birth months	n	Sample (%)	Canadian births (%)
Quarter 1	1980	24.0	23.8
Quarter 2	2097	25.5	25.5
Quarter 3	2345	28.5	26.4
Quarter 4	1811	22.0	24.3
		χ^2^ = 0.39; df = 3; *p* = 0.94	

A Chi-square goodness-of-fit test was performed to compare our samples’ birth month (in quarters) to the average Canadian births by months. The Canadian births are the averages of the percentages of Canadian live births per month between the years 2002 and 2008 [42]. Quarter 1: January–March; Quarter 2: April–June; Quarter 3: July–September; Quarter 4: October–December

The *F*-test values presented in this section represent the main effect of the relative age in quarters or the covariates’ contribution to the model, while the ones shown in Tables 3 and 4 are the corrected model *F*-test values. In Table 3, height of boys and girls was significantly associated with the relative age (*F*(3, 4074) = 57.0, *p* < 0.001 and *F*(3, 4107) = 58.4, *p* < 0.001, respectively). Cohen’s ƒ^2^ effect sizes were considered small for both boys and girls. Moreover, girls’ height findings revealed a dose-response association with the RAE (quarter 1 > quarter 2 > quarter 3 > quarter 4). Girls born in quarter 4 (i.e., born between October to December) were significantly shorter than girls born in all other quarters. There was no significant difference in height between boys born in the last six months of the year (quarters 3 and 4).

**Table 3:** Relative age differences in scores obtained in the Canadian Assessment of Physical Literacy based on children’s month of birth

	Quarter 1 January to March			Quarter 2 April to June			Quarter 3 July to September			Quarter 4 October to December			*F*-test		
	Mean	95% CI		Mean	95% CI		Mean	95% CI		Mean	95% CI				
		LB	UB		LB	UB		LB	UB		LB	UB		*p*	ƒ^2^
Boys															
Height (cm)^a^ (*n* = 4081)	146.2	145.7	146.6	144.6‡	144.1	144.9	142.9‡†	142.5	143.3	142.3‡†	141.9	142.8	563.86^*^	< 0.001	0.04
Weight (kg)^a^ (*n* = 4076)	42.0	41.3	42.6	39.9‡	39.3	40.5	38.9‡	38.3	39.5	38.6‡†	37.9	39.3	175.57^*^	< 0.001	0.02
BMI (kg/m^2^)^a^ (n = 4059)	19.4	19.1	19.6	18.9‡	18.6	19.1	18.8‡	18.6	19.0	18.8‡	18.5	19.0	35.09^*^	0.001	0.004
WC (cm)^a^ (*n* = 4039)	68.9	68.2	69.5	67.4‡	66.8	68.0	67.0‡	66.4	67.6	66.5‡	65.8	67.2	72.28^*^	< 0.001	0.01
Physical Competence domain score^b^ (*n* = 4059)	20.6	20.3	20.8	20.1‡	19.9	20.3	19.8‡	19.6	20.0	19.6‡†	19.3	19.8	277.04^*^	< 0.001	0.01
Daily Behaviour domain score^b^ (*n* = 4059)	18.6	18.1	19.1	18.5	18.0	18.9	18.6	18.1	19.0	19.1	18.6	19.0	12.54^*^	0.26	0.001
Motivation and Confidence domain score^b^ (n = 4059)	12.9	12.7	13.1	12.8	12.6	12.9	12.6	12.5	12.8	12.8	12.6	12.9	5.48^*^	0.42	0.001
Knowledge and Understanding domain score^a^ (*n* = 4100)	12.2	12.0	12.4	12.0	11.9	12.2	11.7‡†	11.5	11.8	11.6‡†	11.5	11.8	78.49^*^	< 0.001	0.01
Total CAPL score^b^ (n = 4059)	64.2	63.4	64.9	63.3	62.6	64.1	62.7‡	61.9	63.4	63.1	62.3	63.9	60.4^*^	0.04	0.002
Girls															
Height (cm)^a^ (*n* = 4114)	146.5	146.1	147.0	144.6‡	144.2	145.1	143.7‡†	143.3	144.1	142.2‡†	141.8	142.7	629.71^*^	< 0.001	0.04
Weight (kg)^a^ (*n* = 4110)	41.5	40.9	42.2	40.4	39.8	41.0	39.5‡	39.0	40.1	38.9‡†	38.2	39.6	201.36^*^	< 0.001	0.01
BMI (kg/m^2^)^a^ (*n* = 4092)	19.1	18.9	19.3	19.0	18.8	19.3	18.9	18.6	19.1	19.0	18.7	19.2	32.93^*^	0.45	0.001
WC (cm)^a^ (*n* = 4076)	67.8	67.2	68.4	67.4	66.7	68.0	66.6‡	66.0	67.1	66.8	66.1	67.6	61.71^*^	0.03	0.002
Physical Competence domain score^b^ (n = 4092)	19.9	19.7	20.2	19.5‡	19.3	19.7	19.2‡	19.0	19.3	18.9‡†	18.7	19.2	276.74^*^	< 0.001	0.01
Daily Behaviour domain score^b^ (n = 4092)	18.5	18.0	18.9	18.2	17.8	18.7	18.0	17.6	18.4	18.5	18.1	19.0	16.08^*^	0.34	0.001
Motivation and Confidence domain score^b^ (n = 4092)	12.5	12.3	12.6	12.3	12.1	12.4	12.2‡	12.0	12.3	12.2‡	12.0	12.3	8.72^*^	0.036	0.002
Knowledge and Understanding domain score^a^ (*n* = 4133)	12.4	12.2	12.6	12.2	12.0	12.3	12.2	12.1	12.4	11.9‡	11.8	12.1	72.83^*^	0.001	0.004
Total CAPL score^b^ (*n* = 4092)	63.3	62.6	63.9	62.1	61.5	62.1	61.5‡	60.9	62.1	61.6‡	60.9	62.3	49.56^*^	0.001	0.004

An analysis of covariance was conducted controlling for multiple covariates described in each model. The *F*-test is shown for the overall corrected model (*p < 0.001). The last two columns, the *p-value and Cohen’s* ƒ^2^ effect sizes, are presented for the main effect of the relative age by quarters

^‡^statistically significant differences between quarter 1 and other quarters

^†^statistically significant differences between quarter 2 and other quarters

^a^controlled for site location, age (whole years) and testing month

^b^controlled for BMI *z*-score, site location, age (whole years) and testing month

*CI* confidence interval, *LB* lower bound, *UB* upper bound, *BMI* body mass index, *WC* waist circumference, *CAPL* Canadian Assessment of Physical Literacy

**Table 4:** Relative age difference, expressed in quarters, and the scores obtained in cardiorespiratory fitness, trunk endurance, upper body strength, flexibility and movement skill assessments for children 8 to 12 years participating in the Canadian Assessment of Physical Literacy

	Boys					Girls				
	PACER (laps) (*n* = 3970)	Timed plank (sec) (*n* = 4044)	Grip strength (kg) (*n* = 4045)	Sit-and-reach (cm) (n = 4044)	CAMSA (*n* = 4011)	PACER (laps) (*n* = 3970)	Timed plank (sec) (*n* = 4080)	Grip strength (kg) (*n* = 4085)	Sit-and-reach (cm) (*n* = 4077)	CAMSA (*n* = 4033)
Quarter 1 (mean [95% CI])	28.1 [27.1–29.0]	63.5 [60.8–66.2]	36.1 [35.5–36.6]	25.4 [24.9–25.9]	32.4 [32.0–32.7]	22.1 [21.4–22.8]	62.7 [60.1–65.2]	34.1 [33.6–34.6]	31.2 [30.7–31.7]	31.4 [31.1–31.8]
Quarter 2 (mean [95% CI])	26.0 [25.1–26.9]‡	62.8 [60.2–65.3]	35.2 [34.7–35.7]	25.6 [25.2–26.1]	31.7 [31.4–32.0]‡	21.2 [20.5–21.9]	62.1 [59.6–64.6]	32.9 [32.5–33.4]‡	30.6 [30.1–31.2]	30.6 [30.3–31.0]‡
Quarter 3 (mean [95% CI])	25.2 [24.4–26.1]‡	62.1 [59.6–64.6]	33.3 [32.8–33.8]‡	25.6 [25.1–26.0]	31.4 [31.1–31.7]‡	20.2 [19.5–20.8]‡	60.2 [57.9–62.6]	32.2 [31.8–32.6]‡	31.1 [30.6–31.5]	30.3 [30.0–30.6]‡
Quarter 4 (mean [95% CI])	25.0 [24.0–25.9]‡	60.7 [57.9–63.4]	33.4 [32.9–33.9]‡	25.2 [24.7–25.6]	31.0 [30.7–31.3]‡†	20.6 [19.9–21.4]‡	60.5 [57.8–63.2]	31.3 [30.8–31.7]‡†	31.0 [30.5–31.6]	29.8 [29.5–30.2]‡†
*F*-test	106.38^*^	53.10^*^	237.40^*^	10.51^*^	109.38^*^	77.7^*^	56.40^*^	320.25^*^	4.34^*^	104.5^*^
*p-*value	< 0.001	0.51	< 0.001	0.53	< 0.001	0.001	0.46	< 0.001	0.43	< 0.001
ƒ^2^	0.01	0.001	0.02	0.001	0.01	0.004	0.001	0.02	0.001	0.01

An analysis of covariance was conducted controlling for BMI *z*-score, site location, age (whole years) and testing month. Quarter 1 represents children born in January to March, quarter 2 is from April to June, etc. The *F*-test is shown for the overall corrected model (^*^p < 0.001). The last two columns, *p*-value and Cohen’s ƒ^2^, are for the main effect of relative age in quarters

^‡^statistically significant differences between quarter 1 and other quarters

^†^statistically significant differences between quarter 2 and other quarters

*CAMSA* Canadian Agility and Movement Skill Assessment, *CAPL* Canadian Assessment of Physical Literacy, *CI* confidence intervals, *PACER* Progressive Aerobic Cardiovascular Endurance Run

Similarly, weight was associated with the relative age differences (*F*(3, 4069) = 20.6, *p* < 0.001 and *F*(3, 4103) = 12.7, *p* < 0.0001, in boys and girls, respectively). The effect sizes for weight were small (ƒ^2^ = 0.02) and negligible (ƒ^2^ = 0.01) in boys and girls, respectively. BMI was significantly associated with the relative age in boys only (*F*(3, 4052) = 5.4, *p* = 0.001 and *F*(3, 4085) = 0.9, *p* = 0.45 in boys and girls, respectively), but the effect size was considered negligible. Among boys and girls, the main effect of relative age was associated with the WC measurement (*F*(3, 4032) = 8.7, *p* < 0.001 and *F*(3, 4069) = 3.1, *p* = 0.03, respectively), but the effect sizes for these associations were also negligible. The covariate age was the largest contributor in the model for height, weight, BMI and WC for both genders (data not shown).

The results for the Physical Competence domain revealed a significant main effect of quarter in both boys and girls (*F*(3, 4051) = 11.8, *p* < 0.0001 and *F*(3, 4084) = 15.7, *p* < 0.0001, respectively), but yielded negligible effect sizes in both boys and girls for the main effect of quarter. However, the covariate BMI *z-*score in the Physical Competence domain score model resulted in large effect sizes in both genders (ƒ^2^ = 0.44 and ƒ^2^ = 0.40 in boys and girls, respectively).

No RAE was observed for the Daily Behaviour domain scores (*F*(3, 4051) = 1.4, *p* = 0.26 and *F*(3, 4084) = 1.1, *p* = 0.34 in boys and girls, respectively). Interestingly, the largest contributor in the model in boys for this domain score was the covariate BMI *z*-score (*F*(1, 4051) = 51.4, *p* < 0.001) while in girls the largest contributor was covariate age (*F*(1, 4084) = 1.1, *p* = 0.34). In terms of effect sizes, the BMI *z-*score covariate in boys was negligible (ƒ^2^ = 0.01) but in the girls’ model the covariate age produced a small effect size (ƒ^2^ = 0.02).

Girls’ Motivation and Confidence domain scores were significantly associated with the relative age (*F*(3, 4084) = 2.9, *p* = 0.04), but the association’s effect size was negligible. Among boys and girls, the Knowledge and Understanding domain scores were also significantly associated with relative age (*F*(3, 4093) = 9.8, *p* < 0.0001 and *F*(3, 4126) = 5.2, *p* = 0.001, respectively). Again, these main effect associations produced negligible effect sizes, while the covariate age in the association between relative age and the Knowledge and Understanding domain scores generated small effect sizes (ƒ^2^ = 0.10 and ƒ^2^ = 0.09 in boys and girls, respectively).

Among both genders, there was a significant main effect of birth month, in quarters, on the overall CAPL scores (*F*(3, 4051) = 2.7, *p* = 0.04 and *F*(3, 4084) = 5.8, *p* = 0.001 in boys and girls, respectively). The effect sizes for the associations between the overall CAPL scores and the relative age were negligible. The covariate BMI *z*-score was the largest contributor in the model, even larger than the age covariate, for the overall CAPL scores in both boys and girls (*F*(1, 4051) = 383.1, *p* < 0.0001, ƒ^2^ = 0.09 and *F*(1, 4084) = 324.7, *p* < 0.0001, ƒ^2^ = 0.08, respectively). The associations between children’s relative age, in quarters, and the specific Physical Competence assessment protocol are presented in Table 4. Despite having a statistically significant corrected model for all the assessments, the main effect of quarters was only statistically significant for children’s cardiorespiratory fitness (PACER shuttle run test), the upper body strength assessment (grip strength), and the movement skill assessment (CAMSA). Among boys and girls, the number of 20-m laps run was associated with the relative age of children (*F*(3, 3962) = 8.6, *p* < 0.0001 and *F*(3, 3962) = 5.8, *p* = 0.001, respectively). However, this significant main effect of relative age for the cardiorespiratory fitness assessment revealed only negligible effect sizes. While the main effect for the cardiorespiratory fitness assessment was negligible, the covariate BMI *z*-score contributed to moderate (ƒ^2^ = 0.16) and small (ƒ^2^ = 0.11) effect sizes in the model in boys and girls, respectively. The significant associations between the movement skills assessment (CAMSA) and the relative age among boys and girls (*F*(3, 4003) = 11.0, *p* < 0.001 and *F*(3, 4025) = 15.8, *p* < 0.001, respectively) also revealed only negligible effect sizes. While effect sizes were negligible for both the cardiorespiratory fitness and the movement skills assessment, the significant association between upper body strength (handgrip strength) and relative age (*F*(3, 4037) = 29.2, *p* < 0.001 and *F*(3, 4077) = 25.1, *p* < 0.001 in boys and girls, respectively) revealed small effect sizes in both genders. Among boys and girls, neither the muscular endurance (timed plank) (*F*(3, 4063) = 0.8, *p* = 0.51 and *F*(3,4072) = 0.87, *p* = 0.46, respectively) nor the flexibility (sit-and-reach) (*F*(3, 4036) = 0.7, *p* = 0.53 and *F*(3,4069) = 0.4, *p* = 0.43, respectively) assessments were associated with the relative age in quarters.

## Discussion

The aim of our study was to quantify the magnitude of the RAE as it relates to CAPL scores (physical literacy) in a large sample of children aged 8 to 12 years. Although we found many significant associations between the domain scores and relative age expressed in quarters, the RAE bias was negligible on the domains and overall CAPL scores. Additionally, we also observed a small relative age association among boys’ and girls’ height and strength measurements. Boys born in the first three months of the year were taller and heavier, and had higher handgrip strength, compared to those born later in the same year. Girls born in the first six months after the school entry cut-off date were taller and had higher handgrip strength compared to their relatively younger peers born in the last six months of the year.

### Anthropometrics

Several studies in sports have hypothesised that the RAE could be attributed to physical advantages based on growth differences [9, 10, 43]. However, this hypothesis is not supported by all the research. In the sport context, a number of studies have observed that relatively older children were significantly taller than those born later in the same year [43–48]; however, others reported no significant associations between relative age difference and anthropometric measurements [49, 50]. Studies examining the association between children’s RAE and fitness or fundamental movement skills assessments outside of a specific sport context have also reported inconsistent RAE results for anthropometric measurements. For instance, Sandercock and colleagues [23] found no association between the RAE and the anthropometrics of their participants. In the present study, we did observe a RAE in children’s height mainly; children born in the first six months of the year were taller than their peers born in the last six months of that same year. The present study’s results are consistent with a previous study [11]. However, these anthropometric advantages seen in the relatively older children did not appear to have much influence on their physical literacy scores.

### Domain scores and overall CAPL scores

Gender differences were observed in the RAE, particularly evident in the Motivation and Confidence domain scores. Boys’ scores were not significantly different based on the main effect of relative age, while girls born in the first three months of the year obtained greater scores in the Motivation and Confidence domain than those born in the last six months of the same year. Previous studies have shown that boys generally display greater self-efficacy and motivation, but also receive greater social support toward physical activities and sports than girls [51, 52]. These gender differences in the psychosocial correlates of physical activity may be partly responsible for the lack of association between the RAE in boys’ Motivation and Confidence scores. Another plausible explanation may involve biological maturation and its association to physical self-concepts [53, 54]. Physical self-concept is considered to be both a determinant and outcome of physical activity, with an increased positive self-concept being positively associated with daily physical activity [55]. In the present study, the adequacy component within the Motivation and Confidence domain was the only component that was significantly associated with the relative age in girls (*F*(3, 4084) = 5.5, *p* = 0.01, ƒ^2^ = 0.004). Adequacy refers to a generalized self-efficacy toward physical activity [38], and being consistently older in school and in sports cohorts may have contributed to the increased level of self-efficacy in the relatively older girls. Both biological maturity status and self-concepts, unmeasured in the present study, could have influenced children’s scores on the Motivation assessment.

Evidence of the RAE in the affective domain is very limited. Thompson and colleagues [16] investigated the association between the RAE of Grade 1 children and self-esteem, and found a positive association between relatively older children and greater self-esteem in school. Although the study was conducted in a classroom setting and in younger children, no gender difference in the relationship between self-esteem and RAE was reported [16]. More research should investigate the association between gender, biological maturity, relative age and different affective outcomes (i.e., motivation, self-efficacy, self-concept, and confidence), since these factors may impact long-term physical activity participation.

Numerous studies have reported on the relationship between a child’s month of birth and academic abilities. Relatively older children tend to consistently score higher on school tests than their relatively younger peers throughout their education [13, 56–60]. In the present study, the RAE results on the association with the Knowledge and Understanding domain scores are consistent with previous studies despite the negligible effect sizes observed. It is important to note that boys born in the first six months of the year scored higher in the Knowledge and Understanding domain than their peers born in the last six months of the year, while girls born in the first three months of the year outscored their peers born in the last three months of the year. In contrast with a recent study [56], the present study’s RAE association with the Knowledge and Understanding domain scores was not attenuated in older children (11 and 12 years old), even though all children completed the same questionnaire. These findings may further substantiate Boardman’s theory [59] that relatively younger children may have different and unmet learning needs than their relatively older peers.

In the present study, while just short of a small effect size, a relative age difference was observed in the Physical Competence domain scores among both genders. Roberts and colleagues [11] have hypothesized that the lower physical fitness they observed among relatively younger children may be due to less daily physical activity. If this were the case, we would have observed a RAE in the Daily Behaviour domain components (i.e., daily step counts and the self-reported number of days a child engages in moderate to vigorous physical activity per week); however, we observed no significant differences between birth months and the individual domain components or the Daily Behaviour domain scores. In fact, the highest Daily Behaviour domain score was seen in boys born in the last three months of the year. Therefore, the amount of daily physical activity does not appear to be a factor in the RAE bias; a more plausible explanation would be the maturation differences and the positive linear relationship between age and performance in this age group [11, 61].

Relative age differences were statistically significant in both boys (two of four domains) and girls (three of four domains); however, these associations yielded negligible effect sizes. Thus, regarding the overall CAPL score, the observed negligible effect sizes were not surprising considering the aggregate nature of the CAPL scoring system. For the domains and the overall CAPL scores, the covariate BMI *z*-score had a greater impact than the relative age, in quarters, for the Physical Competence domain (large effect sizes) and overall CAPL scores (small effect sizes) in both genders. The greater impact observed for the BMI *z*-score covariate could be partially explained by our participants’ mean age and the BMI *z*-score acting as a proxy measure of maturation, as the World Health Organization’s BMI *z*-score are age- (in years and months) and gender- specific [28]. Additionally, the covariate age had a greater impact than the relative age on the Knowledge and Understanding domain (small effect sizes) in both genders, and on the Daily Behaviour domain in girls (small effect sizes).

### Physical competence fitness assessments

Among boys and girls, the strength assessment showed evidence of a RAE bias but the effect sizes were deemed small. The RAE associated with the handgrip strength test is also consistent with a previous study [23]. In this age group, rounded-down age would seem to unfairly compare strength in both genders; this may be related to height and muscle mass differences. It may be worth investigating whether the handgrip strength test is associated with physical literacy or with fitness in this age group, since our results suggest that handgrip strength is highly susceptible to both the children’s anthropometrics (possibly a result of biological maturation variation) and the RAE bias. A nationally representative study conducted in the United States on the association between body weight and strength measurements among children 6 to 15 years of age has observed a positive association between grip strength and weight status, where children who were considered overweight and obese outperformed children of ideal weight [62]. The latter study also reinforced the argument that handgrip strength may be a poor assessment of musculoskeletal fitness in children. Moreover, the poor association between handgrip strength and children’ physical literacy is further discussed by Gunnell and colleagues [63].

Boys born in the first quarter ran significantly more laps compared to their relatively younger peers, while in girls only the comparison between those born in the first quarter and the last six months was significant. While the effect sizes were negligible for the PACER shuttle run test, the more pronounced relative age difference in boys’ cardiorespiratory fitness may be a product of the sample mean age; at 10 years the majority of boys would be prepubescent, while there is an increased likelihood of having more variation in the biological maturation stage of girls [64–66]. The observation of a relative age difference in cardiorespiratory fitness is consistent with previous studies [11, 23, 61].

Among both boys and girls, the CAMSA, a measure of children’s movement skills, was significantly associated with the relative age difference of children. This study’s results showed significant associations between the relative age and the movement skill assessment in both genders, but the main effect of relative age showed negligible effect sizes. These significant associations between RAE and movement skill assessment are consistent with a recent study by Birch and colleagues [22] on the association between the RAE and fundamental movement skills assessments (effect sizes not reported). In contrast with that study, however, we did not observe gender differences in the CAMSA mean scores. The CAMSA produces an overall skill score that could be covering for gender differences in object control versus balance tasks, for example. Birch and colleagues’ observation of gender differences in object control in favour of boys is consistent with findings from a recent systematic review and meta-analysis of the correlates of gross motor skills in children between 3 and 18 years of age [67].

Even though the results for the trunk muscular endurance measurement (timed plank) were not significantly associated with the RAE, the scores did follow the expected pattern for the RAE (quarter 1 > quarter 2 > quarter 3 > quarter 4). However, the expected RAE pattern was not observed for the flexibility assessment (sit-and-reach). The scores were significantly different between boys and girls but no significant differences were observed between whole age groups in girls, whereas younger boys (i.e., ages 8 and 9) scored significantly higher than older boys (data not shown). These observed age and gender differences and scoring patterns in the sit-and-reach are consistent with previous studies observing children’s fitness [68, 69].

Strengths of the present study include the large sample size and the harmonized, validated, population-specific, and age- and gender-normalized protocol that measured physical literacy of children across 11 sites across Canada. Another strength of our study was the high inclusion rate (between 79 and 82% for all samples), despite the need for complete assessment, domain scores and overall CAPL scores. Finally, we believe that this study presents a realistic observation of the RAE bias, in a physical literacy context, as not all studies exploring this bias have reported effect sizes to determine if any differences observed are in fact meaningful.

However, these results need to be interpreted in light of the following limitations. First, because our data are cross-sectional, causality cannot be established. Second, the external generalizability of these findings may be limited due to the nonprobability sampling strategy, which may have produced inflated physical literacy scores. However, our sample birth distribution (in quarters) was representative of the Canadian population birth distribution [42]. Finally, having some information on biological maturation status (e.g., measuring sitting height) would have provided some additional insight that might have helped to confirm some of the theories we advanced to explain our results.

## Conclusions

Collectively, the associations between children’s relative age and the CAPL domain and overall scores produced mainly negligible effect sizes, suggesting that the RAE is not an important factor to consider when assessing the physical literacy of children with the CAPL. In practice, the mean differences observed across birth months, in quarters, were not large enough to warrant an adjustment to the CAPL scores. However, we believe that it is good practice to explore possible RAE bias in new assessment protocols, specifically those that separate children into rounded-down age bands, which may or may not be appropriate for all age groups. As these age bands do not take into account length of time in school, relatively older children are likely to have greater skill development opportunities based on school entry cut-off dates than their peers born later in the year. The lack of RAE findings is positive for the CAPL assessment, since the CAPL’s development was partially informed by schools’ curricula; thus the potential for a RAE warranted exploration. This study is unique as it provides a comprehensive examination of the RAE that assessed the association in affective, cognitive, behavioural, and physical domains of physical literacy in children.

## Additional files

Additional file 1: Canadian Assessment of Physical Literacy scoring system with the scoring weight for each assessment. Adapted from the CAPL Manual for Test Administration. * The “What is Most Like Me” (CSAPPA) questionnaire was developed by Dr. John Hay and is issued in the CAPL with his permission [40]. BMI: body mass index; CAMSA: Canadian Agility and Movement Skill Assessment; CAPL: Canadian Assessment of Physical Literacy; CSAPPA: Children’s Self-Perceptions of Adequacy in and Predilection for Physical Activity; MVPA: moderate to vigorous physical activity; PA: physical activity; PACER: Progressive Aerobic Cardiovascular Endurance Run; WC: waist circumference. (PPTX 68 kb)

Additional file 2: Three-dimensional rendering of the Canadian Agility and Movement Skill Assessment with the list of actions required to be performed by the participants. This rendering was adapted from the CAPL Manual and is not to scale but contains the proper measurements [25]. For this assessment, the participants were evaluated on accuracy of the skills performed and time to complete the assessment. Both time and accuracy are equally important in this assessment in order to reach maximum points. Verbal cues are provided to the participants during the assessment. Two timed/scored trials are needed for the final score, and these are corrected for the age of the participant. Equipment needed: 6 hoops (0.63 m in diameter); 6 cones (of equal size); 1 cardboard target (61 cm in width and 46 cm in height); gym floor tape; 1 soccer ball; and 1 Squelet ball or a soft ball (70 mm in diameter). CAPL: Canadian Assessment of Physical Literacy. (PPTX 414 kb)

## Abbreviations

ANCOVA: analysis of covariance
BMI: body mass index
CAMSA: Canadian Agility and Movement Skill Assessment
CAPL: Canadian Assessment of Physical Literacy
CSAPPA: Children’s Self-Perceptions of Adequacy in and Predilection for Physical Activity
PACER: Progressive Aerobic Cardiovascular Endurance Run
RAE: relative age effect
RBC: Royal Bank of Canada
WC: waist circumference
