# Supplementary material for: Influence of the relative age effect on children’s scores obtained from the Canadian assessment of physical literacy
Source: BMC Public Health. 2018 Oct 2;18(Suppl 2):1040. doi: 10.1186/s12889-018-5895-6 (PMC6167762; doi:10.1186/s12889-018-5895-6)
Supplement: Supplementary file 1 — Canadian Assessment of Physical Literacy scoring system with the scoring weight for each assessment. Adapted from the CAPL Manual for Test Administration. * The “What is Most Like Me” (CSAPPA) questionnaire was developed by Dr. John Hay and is issued in the CAPL with his permission [40]. BMI: body mass index; CAMSA: Canadian Agility and Movement Skill Assessment; CAPL: Canadian Assessment of Physical Literacy; CSAPPA: Children’s Self-Perceptions of Adequacy in and Predilection for Physical Activity; MVPA: moderate to vigorous physical activity; PA: physical activity; PACER: Progressive Aerobic Cardiovascular Endurance Run; WC: waist circumference. (PPTX 68 kb) [file 12889_2018_5895_MOESM1_ESM.pptx]

## Slide 1
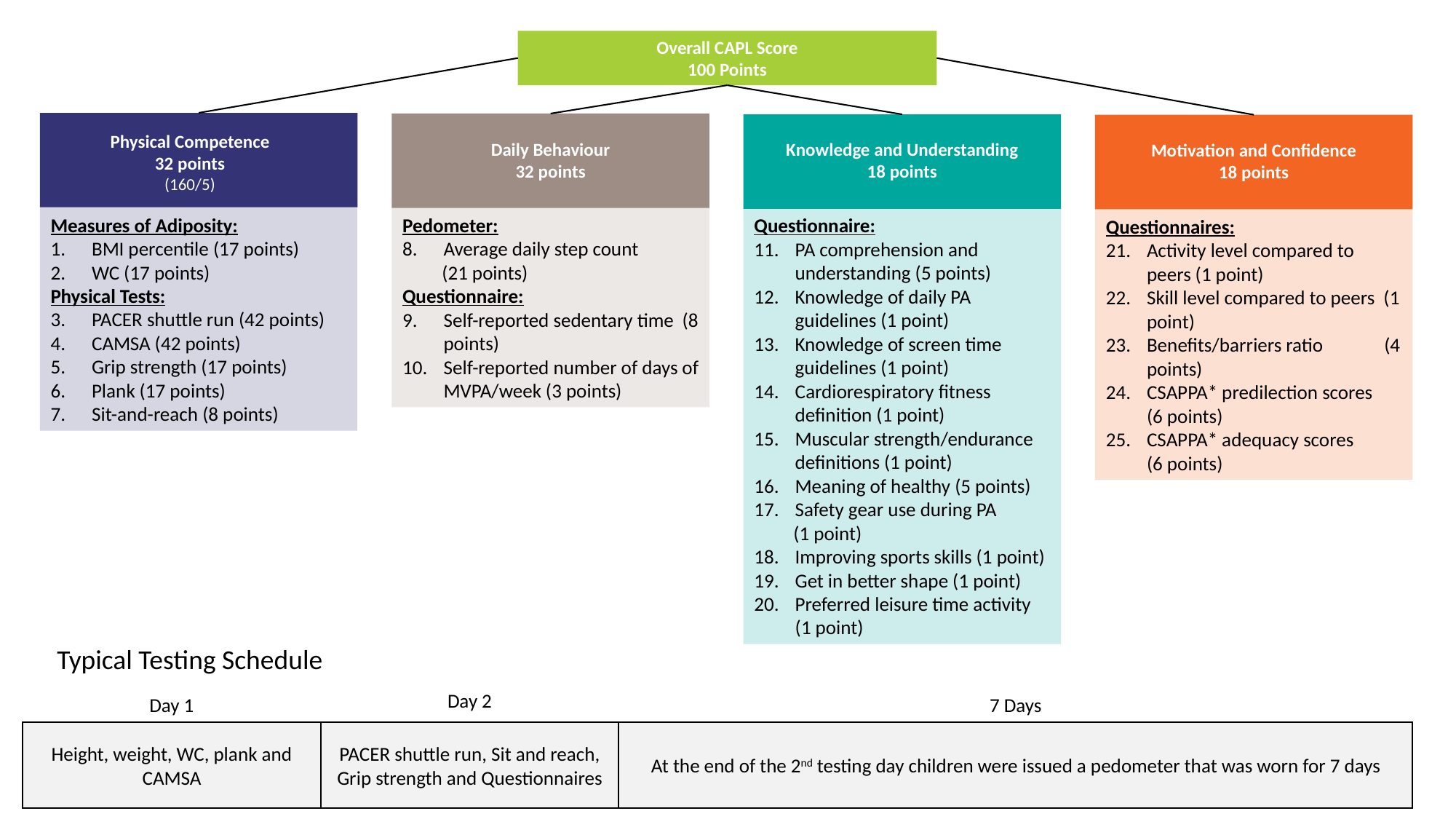

Overall CAPL Score
100 Points
Physical Competence
32 points
(160/5)
Daily Behaviour
32 points
Knowledge and Understanding
18 points
Motivation and Confidence
18 points
Measures of Adiposity:
BMI percentile (17 points)
WC (17 points)
Physical Tests:
PACER shuttle run (42 points)
CAMSA (42 points)
Grip strength (17 points)
Plank (17 points)
Sit-and-reach (8 points)
Pedometer:
Average daily step count
 (21 points)
Questionnaire:
Self-reported sedentary time (8 points)
Self-reported number of days of MVPA/week (3 points)
Questionnaire:
PA comprehension and understanding (5 points)
Knowledge of daily PA guidelines (1 point)
Knowledge of screen time guidelines (1 point)
Cardiorespiratory fitness definition (1 point)
Muscular strength/endurance definitions (1 point)
Meaning of healthy (5 points)
Safety gear use during PA
 (1 point)
Improving sports skills (1 point)
Get in better shape (1 point)
Preferred leisure time activity (1 point)
Questionnaires:
Activity level compared to peers (1 point)
Skill level compared to peers (1 point)
Benefits/barriers ratio (4 points)
CSAPPA* predilection scores (6 points)
CSAPPA* adequacy scores (6 points)
Typical Testing Schedule
Day 2
7 Days
Day 1
Height, weight, WC, plank and CAMSA
PACER shuttle run, Sit and reach, Grip strength and Questionnaires
At the end of the 2nd testing day children were issued a pedometer that was worn for 7 days
