# Supplementary material for: Influence of the relative age effect on children’s scores obtained from the Canadian assessment of physical literacy
Source: BMC Public Health. 2018 Oct 2;18(Suppl 2):1040. doi: 10.1186/s12889-018-5895-6 (PMC6167762; doi:10.1186/s12889-018-5895-6)
Supplement: Supplementary file 2 — Three-dimensional rendering of the Canadian Agility and Movement Skill Assessment with the list of actions required to be performed by the participants. This rendering was adapted from the CAPL Manual and is not to scale but contains the proper measurements [25]. For this assessment, the participants were evaluated on accuracy of the skills performed and time to complete the assessment. Both time and accuracy are equally important in this assessment in order to reach maximum points. Verbal cues are provided to the participants during the assessment. Two timed/scored trials are needed for the final score, and these are corrected for the age of the participant. Equipment needed: 6 hoops (0.63 m in diameter); 6 cones (of equal size); 1 cardboard target (61 cm in width and 46 cm in height); gym floor tape; 1 soccer ball; and 1 Squelet ball or a soft ball (70 mm in diameter). CAPL: Canadian Assessment of Physical Literacy. (PPTX 414 kb) [file 12889_2018_5895_MOESM2_ESM.pptx]

## Slide 1
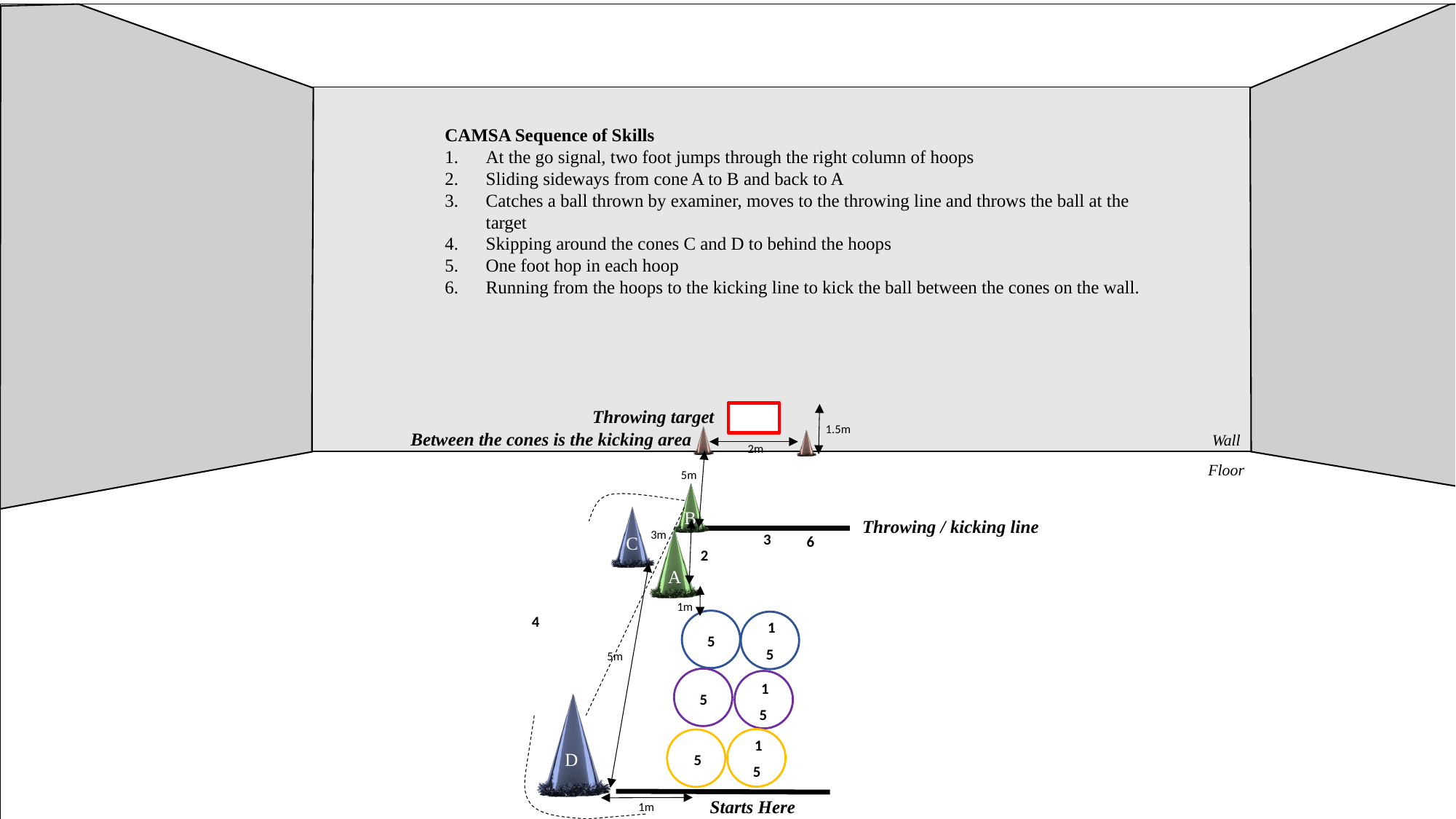

CAMSA Sequence of Skills
At the go signal, two foot jumps through the right column of hoops
Sliding sideways from cone A to B and back to A
Catches a ball thrown by examiner, moves to the throwing line and throws the ball at the target
Skipping around the cones C and D to behind the hoops
One foot hop in each hoop
Running from the hoops to the kicking line to kick the ball between the cones on the wall.
Throwing target
1.5m
Between the cones is the kicking area
Wall
2m
Floor
5m
B
Throwing / kicking line
3m
3
6
C
2
A
1m
4
1
5
5
5m
1
5
5
1
5
D
5
Starts Here
1m
